# Does Transient Receptor Potential Vanilloid Type 1 Alleviate or Aggravate Pathological Myocardial Hypertrophy?

**DOI:** 10.3389/fphar.2021.681286

**Published:** 2021-05-10

**Authors:** Qiqi Yan, Jun Tang, Xin Zhang, Liuyang Wu, Yunyi Xu, Lihong Wang

**Affiliations:** ^1^ The Second Clinical Medical College, Zhejiang Chinese Medical University, Hangzhou, China; ^2^ Department of Cardiovascular Medicine, Zhejiang Provincial People’s Hospital, People’s Hospital of Hangzhou Medical College, Hangzhou, China

**Keywords:** transient receptor potential vanilloid type 1, myocardial hypertrophy, cardiac structural remodelling, myocardial fibrosis, inflammation

## Abstract

Transient receptor potential vanilloid type 1 (TRPV1) is a non-selective cation channel, which is involved in the endogenous stress adaptation mechanism for protection of the heart as well as the occurrence and development of some heart diseases. Although the effect of activation of the TRPV1 channel on different types of non-neural cells in the heart remains unclear, most data show that stimulation of sensory nerves expressing TRPV1 or stimulation/overexpression of the TRPV1 channel has a beneficial role in heart disease. Some studies have proven that TRPV1 has an important relationship with pathological myocardial hypertrophy, but the specific mechanism and effect are not clear. In order to help researchers better understand the relationship between TRPV1 and pathological myocardial hypertrophy, this paper aims to summarize the effect of TRPV1 and the related mechanism in the occurrence and development of pathological myocardial hypertrophy from the following three points of view: 1) role of TRPV1 in alleviation of pathological myocardial hypertrophy; 2) role of TRPV1 in aggravation of pathological myocardial hypertrophy; and 3) the point of view of our team of researchers. It is expected that new therapies can provide potential targets for pathological myocardial hypertrophy.

## Introduction

### Transient Receptor Potential Vanilloid Type 1

Transient receptor potential cation channel, subfamily V, member 1 (TRPV1), is a widely reported non-selective cation channel. TRPV1 can be activated or inhibited by a variety of physical or chemical substances. Its agonists, such as capsaicin, are the most commonly used as analgesic agents. Its antagonists include capsazepine (Bevan et al., 1992; Walpole et al., 1994; Szallasi and Blumberg, 1999). After TRPV1 activation, nerve permeability to calcium ions increases and intracellular calcium ion levels rise, leading to nerve stimulation. This stimulation can be conducted upward to the central nervous system to form an autonomic reflex and can prompt the sensory nerve terminals locally to release neurotransmitters, including somatostatin (SOM), calcitonin gene-related peptide (CGRP), substance P(SP), and other neuropeptides (Levite et al., 1998; Okajima and Harada, 2006).

The TRPV1 receptor is a transducer and molecular integrator of peripheral noxious stimulation (Cao et al., 2013; Sisignano et al., 2014). TRPV1 is expressed in a variety of cells and is also found in the regions of the cardiovascular system (Zhong and Wang, 2009), such as the myocardium and coronary system, having dense capsaicin sensitive sensory innervation and TRPV1 expression.

### Transient Receptor Potential Vanilloid Type 1 and Myocardial Hypertrophy

Studies have reported that multiple neurotransmitters released by TRPV1 can directly regulate cardiac function (Wang and Wang, 2005). Previous studies have shown that TRPV1 plays a protective role in cardiac ischaemic stress (Wang and Wang, 2005; Zhong and Wang, 2007; Huang et al., 2009), and an anti-inflammatory role in hypertension (Wang, 2008). A link between TRPV1 and myocardial hypertrophy in mice was also proven (Huang et al., 2009; Thilo et al., 2010; Buckley and Stokes, 2011). In addition to TRPV1, many TRPV channels play a key role in heart functioning. For example, the activation of TRPV2 is a prerequisite for normal cardiac pumping function (Koch et al., 2018).

Myocardial hypertrophy occurs when the preload or afterload of the heart increases, and the size of the heart and single cardiac muscle cells increases to maintain perfusion of the peripheral organs. Myocardial hypertrophy includes reversible physiological and irreversible pathological hypertrophy. Pathological hypertrophy is often accompanied by adverse cardiovascular events.

A correlation between TRPV1 and myocardial hypertrophy is known, but the role of TRPV1 in the pathogenesis of myocardial hypertrophy and the underlying molecular mechanisms remain unclear. Therefore, in this review, we aim to discuss the current research on the role of TRPV1 in pathological myocardial hypertrophy to provide a reference for the development of drugs for the treatment of pathological hypertrophy in the future.

Limited studies in literature have investigated the role of TRPV1 in the pathogenesis of myocardial hypertrophy and the underlying molecular mechanisms and the findings are not uniform. All relevant studies have been mentioned and discussed in this review (Table 1).

**TABLE 1:** Summary for the role of TRPV1 in Pathological Myocardial Hypertrophy.

Experimental model		Treatment to modulate TRPV1	Location of TRPV1	Effects of treatment	Role of TRPV1 on pathological myocardial hypertrophy	References
In vivo	WT or TRPV1-/- mice on high-salt diet	Genetic deletion; dietary capsaicin	In the heart	TRPV1 activation upregulated PPAR-δ and UCP2 protein expression and decreased iNOS production; relieved oxidative/nitrotyrosine stress	Beneficial	Gao et al. (2014)
In vitro	H9C2 cells	Capsaicin	H9c2 cells	Increased PPAR-δ expression in cardiomyocytes		
In vivo	WT or TRPV1-/- mice on high-salt diet	Genetic deletion; dietary capsaicin	In both the mitochondria and cytoplasm of cardiomyoblasts	TRPV1 activation protected mitochondria from dysfunction by increasing cardiac mitochondrial sirtuin 3 expression, the proficiency of Complex I OXPHOS, ATP production and Complex I enzyme activity	Beneficial	Lang et al. (2015)
In vitro	H9C2 cells	Capsaicin		Markedly enhanced the expressions of TRPV1 and sirtuin 3; sirtuin 3 regulated the expression of NDUFA9 and benefited mitochondrial function		
In vivo	WT or ETA-/- mice in a low-temperature environment of 4°C	TRPV1 agonist SA13353; capsazepine	Cardiomyocytes	SA13353 attenuated, while capsazepine mimicked cold stress- or ET-1-induced cardiac dysfunction	Beneficial	Zhang et al. (2012)
In vivo	WT or TRPV1-/-mice with AB	Genetic deletion; dietary capsaicin	Cardiomyocytes	TRPV1 activation down-regulated TGF-β1/Smad2/3 signaling; inhibited cardiac fibroblast proliferation	Beneficial	Wang et al. (2014)
In vivo	WT or TRPV1-/-mice with TAC	Genetic deletion; activated by the up-regulation of TNFα after TAC	Expressed in sensory nerves	TRPV1 activation increased the release of CGRP, thereby reducing the secretion of inflammatory mediators, including TNFα and IL-6	Beneficial	Zhong et al. (2018)
In vivo	PP2Ac α transgenic mice, TRPV1-/- mice or WT mice	Genetic deletion; transgenic mice elevate the expression of TRPV1 transcripts	In the epicarp of the heart	An increased transmembrane calcium influx through TRPV1 channels may aggravate excitation–contraction coupling, apoptosis of cardiac cells, and finally cardiac remodeling	Detrimental	Thilo et al. (2010)
In vivo	WT or TRPV1-/-mice with TAC	Genetic deletion	Cardiomyocytes	Increased the expression of ANP and TGFbeta in the plasma	Detrimental	Buckley and Stokes (2011)
In vivo	C57 mice with TAC	TRPV1 antagonists	Cardiomyocytes	TRPV1 is a physical component of the natriuretic peptide A, cGMP, and PKG signalling complex; drug inhibition of TRPV1 could reverse pre-established hypertrophy by TAC	Detrimental	Horton et al. (2019)
In vivo	Mice with TAC	Intrathecal RTX administration	Expressed in the dorsal horn	Abolished TRPV1 expression and reduced over-activated CSNA, thereby preventing myocardial hypertrophy, fibrosis, and apoptosis	Detrimental	Wang et al. (2019)
In vivo	C57 mice in a low-temperature environment of 4°C after aortic constriction	TRPV1 antagonist SB366791	In left ventricular tissues	Decreased mitochondrial damage and ROS production; changed autophagy-related protein	Detrimental	Lu and Xu (2013)
In vivo	C57 mice with TAC	Capsazepine	In the heart	Capsazepine reduced the expression of ornithine decarboxylase and cardiac polyamine levels	Detrimental	Chen et al. (2016)
In vitro	Neonatal rat cardiomyocytes and H9C2 cells	Capsaicin or ANA (endogenous activator anandamide)	Neonatal rat cardiomyocytes and H9C2 cells	Increased the expression of ANP mRNA, and intracellular Ca^2+^ level; increased the cell volume through MAPK signalling pathway		
		Silenced by small interfering RNA (siRNA); capsazepine		Eliminated or reversed the situations mentioned above		

## Role of Transient Receptor Potential Vanilloid Type 1 in Alleviating Pathological Cardiac Hypertrophy

Many experimental studies have shown that TRPV1 plays a protective or preventive role in the occurrence and development of pathological myocardial hypertrophy. In these studies, TRPV1 was observed to play a beneficial role in the model of pathological cardiac hypertrophy, such as protecting mitochondrial function and reducing anti-inflammatory response.

### Upregulation of Peroxisome Proliferation-Activated Receptors δ and Reduction of Oxidative Stress

Several studies have shown that myocardial hypertrophy and fibrosis induced by a high-salt diet are related to an increase in reactive oxygen species (ROS). In addition, PPAR-δ activation plays a protective role in atherosclerosis, myocardial injury, and myocardial hypertrophy (Graham et al., 2005; Sheng et al., 2008; Takata et al., 2008). It has been reported that the expression of PPAR-δ decreased and the production of 3-nitrotyrosine increased in mice fed with a high-salt diet, which eventually led to myocardial hypertrophy. In contrast, dietary capsaicin significantly increased the expression of PPAR-δ in the left ventricular muscle of wild-type (WT) mice but had no effect on TRPV1 knockout (TRPV1-/-) mice. Dietary capsaicin intake activates the TRPV1 channels, reduces the level of oxidative stress and expression of fibrotic and hypertrophic proteins, and prevents the development of congestive heart failure (Wang et al., 2014). This beneficial effect may be due to the upregulation of PPAR-δ expression mediated by TRPV1, thus protecting the heart from oxidative stress-induced myocardial damage (Gao et al., 2014). Furthermore, this was the first study to prove that a chronic capsaicin diet can improve the condition of left ventricular hypertrophy caused by a high-salt diet (Gao et al., 2014).

### Improvements in Mitochondrial Respiratory Function and Protection of Mitochondrial Integrity

It is well known that ATP production through mitochondrial respiration is the main source of energy for myocardial cells (Abel and Doenst, 2011). Clinical trials and experimental studies have shown that mitochondrial dysfunction can lead to cardiac hypertrophy (Rosca and Hoppel, 2010). Complex I is an important respiratory enzyme in the mitochondrial respiratory electron transport chain. A long-term decrease in its activity can lead to a decrease in mitochondrial ATP production that can damage the myocardial contractile function, resulting in myocardial hypertrophy.

A recent study (Lang et al., 2015) found that both TRPV1 knockout and a chronic high-salt diet can reduce the proficiency of mitochondrial Complex I oxidative phosphorylation (OXPHOS), expression of cardiac mitochondrial sirtuin 3, production of ATP, and activity of Complex I enzyme. These affected processes can impair the function of cardiac mitochondria, which can directly increase the severity of cardiac hypertrophy. However, the situation above was observed to improve with capsaicin intake in WT mice, but not in TRPV1-/- mice. This result proves that chronic dietary capsaicin intake helps in reducing high-salt-induced mitochondrial dysfunction and cardiac hypertrophy and is mediated by TRPV1(Lang et al., 2015).

In addition, mitochondrial dysfunction triggers a series of adverse cellular events, resulting in improper utilisation of intracellular calcium (Ca^2+^) and contractile dysfunction (Ren et al., 2010; Zhang et al., 2011). Some researchers have observed that TRPV1 agonists failed to induce cold exposure-induced mitochondrial depolarisation, which was beneficial for promoting the protective effect of TRPV1 on mitochondria under cold stress in endothelin-A receptor knockout (ETA-/-) mice. The TRPV1 antagonist, capsazepine, mimicked cold stress-induced cardiac anomalies, whereas the TRPV1 agonist, SA13353, attenuated it (Zhang et al., 2012).

### Improvement of Collagen Degradation and Inhibition of Myocardial Fibrosis

Transverse aortic constriction (TAC) is also a common method of inducing an acute pressure overload model, such as suprarenal aortic banding (AB). Cardiac diastolic dysfunction induced by TAC may be caused by myocardial fibrosis, which may aggravate the development of left ventricular remodelling and diastolic dysfunction and heart failure (Wang et al., 2016). However, the study by Wang et al. (2014) shows that capsaicin could inactivate the upregulation of transforming growth factor β, connective tissue growth factor, and the phosphorylation of Smad2/3 induced by pressure overload in WT mice but had no effect on TRPV1-/- mice. In addition, capsaicin can reduce the overexpression of MMP-2, MMP-9, and MMP-13 induced by pressure overload in WT mice, which are responsible for the degradation of collagen. However, these effects of capsaicin were not present in TRPV1-/- mice. Capsaicin also attenuated angiotensin II-induced proliferation of mouse cardiac fibroblasts with the TRPV1 channel (Wang et al., 2014). In a word, dietary capsaicin has protective effects on myocardial hypertrophy and fibrosis in pressure overload mice through TRPV1.

### Anti-Inflammatory Responses

Evidence suggests that microRNA-155 expression in macrophages promotes inflammation, hypertrophy, and heart failure under a TAC-induced pressure overload (Heymans et al., 2013). Indeed, the TAC can effectively stimulate cardiac hypertrophy, leading to an inflammatory reaction in the experimental environment. Several studies have shown that inflammation plays a crucial role in the development of cardiac fibrosis (Gupta et al., 2012; Doltra et al., 2013). Interleukin 6 (IL-6), tumour necrosis factor α (TNF α), activation of nuclear factor-kB (NF-κB), nuclear factor of activated T-cells (NFAT), and activator protein 1 signalling pathways are involved in the inflammatory response (Singh et al., 1996; Uozumi et al., 2001; Li et al., 2004; Sancho et al., 2004; Sun et al., 2007). For example, IL-6 is involved in myocardial fibrosis, hypertrophy, and diastolic dysfunction (Melendez et al., 2010). Activation of the NF-κB signalling pathway plays an important role in the pathogenesis of cardiac hypertrophy and development of heart failure (Zelarayan et al., 2009; Gordon et al., 2011; Hamid et al., 2011).

A recent study (Zhong et al., 2018) found that TRPV1 regulates the inflammatory process to protect the heart from pressure overload-induced hypertrophy and inflammation. It is suggested that the upregulation of TNF α after the TAC may activate TRPV1 and increase the expression of TRPV1 and release of CGRP, thereby reducing the secretion of inflammatory mediators. In addition, TAC can significantly increase the level of anti-inflammatory cytokine IL-10 in WT mice, which could inhibit the increase in IL-6 and TNF α, but not in TRPV1-/- mice (Demirbilek et al., 2004; Zhong et al., 2018). Marshall et al. found that greater IL-10 was detected in the plasma of WT mice fed with high-fat food, but no significant change was observed in TRPV1-/- mice, which is consistent with the results of this study (Marshall et al., 2013). Moreover, TRPV1 activators such as endovanilloids can inhibit the activation of NF-κB, NFATs, and activator protein 1 signalling pathways (Singh et al., 1996; Demirbilek et al., 2004; Sancho et al., 2004). In conclusion, the TRPV1 gene promotes the above anti-inflammatory process, thus alleviating the pressure overload-induced cardiac hypertrophy and playing a cardioprotective role.

## Role of Transient Receptor Potential Vanilloid Type 1 in Aggravating Pathological Cardiac Hypertrophy

Contrary to the above study findings, the results of the following experimental studies represent another point of view. The results suggest that the absence of TRPV1 or TRPV1 antagonists can prevent or alleviate pathological cardiac hypertrophy. For example, it has been confirmed that in heart failure, Ca^2+^ influx is caused by continuous increase in intracellular Ca^2+^ concentration through the L-type calcium channels and the activation of the *ß*-adrenergic receptors (Horiuchi-Hirose et al., 2011; Timofeyev et al., 2013). Moreover, one study has shown that Ca^2+^ CaMKII is involved in the activation of TRPV1-induced cardiac hypertrophy (Chen et al., 2016).

### Relationship With the Overexpression of Protein Phosphatase 2A

Protein phosphatase 2A (PP2A) plays a key role in excitable cellular signalling (Bhasin et al., 2007). It can dephosphorylate phosphatase, an intrinsic membrane protein of the myocardial sarcoplasmic reticulum, thereby reducing the activity of sarcoplasmic reticulum calcium ATPase. This results in the decrease of intracellular storage filling, thereby reducing the positive muscle strength of the heart. One study observed that the expression of TRPV1 protein in PP2Ac α transgenic mice was also significantly higher than that in WT mice. Moreover, compared with TRPV1-/- mice, PP2Ac α transgenic mice showed more cardiac fibrosis (Thilo et al., 2010). This suggests that TRPV1 expression is associated with cardiac hypertrophy in this new genetic model of impaired cardiac function caused by overexpression of the cardiac-specific protein phosphatase 2A catalytic subunit (Thilo et al., 2010).

### Upregulation of Atrial Natriuretic Peptide and TGFbeta Expression

Cardiac compliance depends on the structural properties of myocardium and connective tissue (Swynghedauw, 1999). Cardiac connective tissue is an important factor in cardiac compliance (Spinale, 2007). Cell death is another important factor in cardiac remodelling (Swynghedauw, 1999; Zhao et al., 2016). Buckley et al. used the TAC method to establish an acute pressure overload model, and their results showed that the expression of atrial natriuretic peptide (ANP) and TGFbeta [as a late marker of cardiac hypertrophy (Lijnen and Petrov, 1999a; Lijnen and Petrov, 1999b)] in the plasma of WT mice was higher than that in TRPV1-/- mice (Buckley and Stokes, 2011). Moreover, the expression of TRPV1 was upregulated in WT mice with myocardial hypertrophy after surgery, and the cardiac function was worse than that in mice without functional TRPV1. In addition, the markers of hypertrophy, fibrosis, and apoptosis in TRPV1-/- mice were lower than those in WT mice (Buckley and Stokes, 2011).

### Transient Receptor Potential Vanilloid Type 1 as a Physical Component of Natriuretic Peptide A, cGMP, and PKG Signalling Complex

Horton and other scholars indicated that a TRPV1 antagonist can be used to overcome the loss of cardiac function. And they also considered that a TRPV1 antagonist might provide a new treatment strategy for cardiac hypertrophy and heart failure (Horton et al., 2013; Horton et al., 2019).

ANP can activate the signal pathway of cardiac hypertrophy. Myocardial fibrosis and ANP are considered as markers of cardiac hypertrophy (Ho et al., 2012; Lijnen et al., 2012). In that study, Horton administered TRPV1 antagonist orally to assess any reversal of loss of function associated with pressure overload cardiac hypertrophy (Horton et al., 2019). They found that TRPV1 is a physical component of the natriuretic peptide A, cGMP, and PKG signalling complex, interacting with natriuretic peptide receptor 1 (NPR1), and upon binding with its ligand, natriuretic peptide A (NPPA), TRPV1 activation was subsequently suppressed through production of cGMP and PKG-mediated phosphorylation of the channel. Among them, cGMP and PKG have a recognised relationship with heart health (Takimoto, 2012; Janssen et al., 2013; Vemula et al., 2014; Lee et al., 2015). Drug inhibition of TRPV1 can inhibit ventricular and cardiomyocyte hypertrophy induced by aortic coarctation in mice with chronic pressure overload and improve cardiac function (Horton et al., 2019).

### Effects on the Function of the Sympathetic Nerve

In addition, resin toxin (RTX) is a selective TRPV1 receptor agonist, which can eliminate TRPV1 + primary sensory afferent reflexes and inactivate cardiac sympathetic afferent reflexes for a long time (Szallasi and Blumberg, 1989). Evidence suggests that sympathetic activity is always over activated during cardiac hypertrophy (Triposkiadis et al., 2009). Therefore, an effective way to prevent the hypertrophic heart from developing into heart failure is by blocking sympathetic hyperactivity. In one study, mice were intrathecally injected with RTX (2 μg/10 μl) into the T2/T3 space 5 days before TAC (Wang et al., 2019). Cardiac sympathetic activity (CSNA) and cardiac structure and function were measured 8 weeks after TAC. The results showed that intrathecal injection of RTX could inhibit the expression of TRPV1 in the dorsal horn of TAC rats, reduce the over-activated CSNA, and improve the cardiac compliance. In addition, RTX could prevent myocardial hypertrophy, fibrosis, and apoptosis induced by TAC and reduce the expression of apoptotic proteins. These results suggest that local chemical ablation of TRPV1+ in the afferent spinal cord can protect the heart from the effects of pressure overload on cardiac remodelling and dysfunction (Wang et al., 2019).

### Promoting Autophagy in a Reactive Oxygen Species- and AMPK-dependent Manner

In contrast to the results of Zhang’s study (Zhang et al., 2012), Lu’s study (Lu and Xu, 2013) suggested that cold exposure aggravates the heart fat caused by pressure overload through TRPV1. Severe cold exposure and pressure overload can cause oxidative stress and pathological changes in the heart. Continuous cold exposure can reduce the antioxidant capacity of myocardial cells and promote the production of ROS (Hong et al., 2008). In addition, mitochondria are considered the main target of oxidative damage under stress (Dai and Rabinovitch, 2011). There is evidence that TRPV1 promotes autophagy in a ROS- and AMPK-dependent manner (Farfariello et al., 2012). As it is known that capsaicin, a natural TRPV1 ligand, can activate the cell fuel signalling molecule AMPK (Farfariello et al., 2012). Lu et al. put WT mice in a low-temperature environment of 4°C for 4 weeks after aortic coarctation to observe the changes. The results showed that continuous cold stress aggravated myocardial hypertrophy and systolic dysfunction in mice. At the same time, TRPV1 expression in WT mice was upregulated; mitochondrial damage was increased; autophagy-related protein, including the phosphorylation of AMPK and mTOR, and the expression of LC3B and p62, was changed; and ROS production was increased. These effects were counteracted by SB366791, a TRPV1 receptor antagonist (Lu and Xu, 2013). In conclusion, these data suggest that cold exposure aggravates myocardial hypertrophy and contraction defects induced by pressure overload through TRPV1 and autophagy-dependent mechanisms (Zhang et al., 2012).

### Activation of the Mitogen-Activated Protein Kinase Signalling Pathway and Effect on Intracellular Polyamine Expression

There is evidence that Ca^2+^ plays a key role in the long-term structural remodelling or degeneration of the heart. Previous studies have shown that increasing Ca^2+^ influx can activate the calmodulin signalling pathway, resulting in increased expression of ANP and MMP9 (Kuwahara et al., 2006). TRPV1 also has the function of creating a Ca^2+^ permeability channel, which can lead to an increase in the level of intracellular Ca^2+^ (Caterina et al., 1997; Nishida and Kurose, 2008). Therefore, researchers have studied this mechanism. In cultured cardiomyocytes, the activation of TRPV1 increased the cell volume, expression of ANP mRNA, and intracellular Ca^2+^ level, which could be reversed by the TRPV1 antagonist capsazepine. The expression of phosphorylated calmodulin-dependent protein kinase IID and mitogen-activated protein kinase (MAPK) were increased in TRPV1 agonist capsaicin-treated cardiomyocytes. Capsaicin increased the expression of ornithine decarboxylase, a key enzyme in polyamine synthesis in cardiomyocytes. TRPV1 antagonist can decrease the expression of ornithine decarboxylase and cardiac polyamine in mice treated with transverse artery contraction. This study suggests that the MAPK signalling pathway and intracellular polyamines are important in TRPV1-induced cardiac hypertrophy (Chen et al., 2016).

## Conclusion

Myocardial hypertrophy increases the work of myocardial cells through the thickening of the ventricular wall, so as to maintain the heart pumping blood to meet the needs of peripheral organs under the condition of increased load. This process is considered an adaptive and compensatory response. Physiological hypertrophy has normal or enhanced systolic function, and the structure and tissue of the heart structure are also normal, which can help in heart functioning (Maillet et al., 2013); while pathological hypertrophy is associated with cardiac structural remodelling and myocardial fibrosis, which can lead to adverse cardiovascular events, including heart failure and death (Roger et al., 2011). Pathological cardiac hypertrophy should be viewed in the context of human clinical data showing that the hazard ratio for all-cause mortality increases by 39% for every 10% reduction in ejection fraction [albeit below 45% (Solomon et al., 2005)] and that the estimated risk of a cardiac event is doubled for every 10% decrease in ejection fraction (Dakik et al., 1996).

The experimental studies discussed above represent the most recent experimental studies to explore the relationship between TRPV1 and pathological myocardial hypertrophy. Careful comparison of their conclusions showed that there are many consistent results as well as many variations in results. This phenomenon indicates the complex role of TRPV1 under pathophysiological conditions. Differences may be the result of many factors. TRPV1 gene knockout mice systematically delete the TRPV1 receptor in themselves, resulting in systematic TRPV1 deletion from birth, which leads to developmental and compensatory changes (Wang and Wang, 2005). However, the use of TRPV1 receptor antagonists, such as capsazepine, is obviously different from gene knockout technology. The antagonists are used to block the TRPV1 receptor in an acute manner, and there is no time to produce a compensatory response. RTX, a selective receptor agonist of TRPV1, not only deleted TRPV1 receptor in cardiac afferent cells but also damaged nerve endings expressing TRPV1. The cardiac sympathetic afferent terminals expressing TRPV1 also express many other sensory receptors, such as tachykinin and purinergic receptors. RTX also damages these sensory receptors after damaging the cardiac afferent terminals expressing TRPV1 (Zahner et al., 2003). TAC is a common way to create a cardiac hypertrophy model. In the experimental environment, TAC is an effective stimulation to produce cardiac hypertrophy quickly, rather than a gradual onset (Buckley and Stokes, 2011). A high-salt diet and other stimulation methods may induce a chronic onset, so there is a possibility of compensation. In conclusion, we believe that different treatments of TRPV1 may or may not achieve the same cardiovascular effects.

Our previous studies have shown that TRPV1 deficiency impairs post-ischaemic recovery and increases inflammation and cardiac remodelling in isolated perfused hearts after myocardial infarction (Wang and Wang, 2005). There is a body of evidence indicating that inflammation plays a crucial role in the development of cardiac fibrosis (Gupta et al., 2012; Doltra et al., 2013). Our previous studies have shown that TRPV1 may protect the heart from injury by increasing the release of substance P, which may play a role in developing left ventricular end-diastolic pressure and left ventricular pressure, increasing coronary blood flow and improving cardiac function. In addition, we also demonstrated that the deletion of this receptor may enhance the function of the NK1 receptor to regulate the heart to avoid injury, at least to a certain extent, by enhancing the function of the NK1 receptor. This suggests that the long-term deletion of the gene can cause a non-TRPV1-dependent compensatory mechanism, which may play a protective role in the heart. This conclusion also partly explains the reasons for the above findings (Wang and Wang, 2005).

Capsaicin is the active ingredient of capsicum, which is considered to have anti-inflammatory effect (Kim et al., 2003). Our previous study is consistent with the anti-inflammatory effect proposed by Zhong (Zhong et al., 2018). In addition, more studies have shown the multiple benefits of capsaicin in activating the TRPV1 receptor. Evidence has confirmed that TRPV1 agonists (such as capsaicin) have beneficial cardiovascular effects through neurotransmitter release and inhibition of platelet aggregation (Luo et al., 2011). Dietary capsaicin has been shown to improve several cardiovascular diseases, including obesity, dyslipidaemia, hyperglycaemia, hypertension, and atherosclerosis (Kang et al., 2010; Hollis and Wang, 2013). The effect of capsaicin mainly depends on its receptor TRPV1.

Although several TRPV1 agonists are commonly used for pain relief, the potential of TRPV1 analogues in the treatment of cardiovascular diseases is just emerging. Therefore, it is crucial to understand the role of TRPV1 so that medical professionals can use the existing and unique TRPV1 agonist or antagonist pharmacopoeia to treat pathological cardiac hypertrophy and other diseases. Further studies are needed to confirm TRPV1 as a potential cardiac target. Our team plans to conduct more studies in the future to further explore the relationship between TRPV1 and the occurrence and development of pathological cardiac hypertrophy and the internal mechanisms.
